# Acute Hypothenar Hammer Syndrome With Digital Ischemia in a Professional Rakugo Performer

**DOI:** 10.1016/j.jhsg.2025.100761

**Published:** 2025-06-03

**Authors:** Daiki Saito, Norie Kodera, Makoto Hirao

**Affiliations:** ∗ Department of Orthopaedic Surgery, Nippon Medical School, Tokyo, Japan

**Keywords:** Digital ischemia, Hand surgery, Hypothenar hammer syndrome, Occupational injury, Pseudoaneurysm

## Abstract

We report a rare case of hypothenar hammer syndrome in a 59-year-old professional Rakugo performer who developed acute digital ischemia in the right hand. Imaging revealed a thrombosed ulnar artery pseudoaneurysm, which was surgically resected and reconstructed using a vein graft. The patient had a history of blunt hand trauma over a decade earlier but remained asymptomatic until sudden onset of ischemia following excessive alcohol intake. This case highlights the potential for delayed yet acute presentation of hypothenar hammer syndrome and underscores the importance of recognizing occupational microtrauma and systemic triggers in vascular hand conditions.

Hypothenar hammer syndrome (HHS) is a rare vascular disorder caused by repetitive trauma to the ulnar artery at the hypothenar eminence, often resulting in arterial wall damage, pseudoaneurysm formation, and distal embolization. It is typically seen in individuals engaged in occupations or activities involving repetitive impact or vibration to the ulnar side of the hand, and most cases follow a chronic course with gradual symptom development. We report a unique case of HHS in a professional Rakugo performer—a traditional Japanese storyteller—who likely experienced chronic hypothenar microtrauma through repeated use of a folding fan (sensu) during performances. Remarkably, the patient developed acute digital ischemia more than 10 years after an initial hand injury, following a period of heavy alcohol intake. This case highlights the potential for delayed yet sudden onset of ischemia in HHS and emphasizes the importance of recognizing atypical occupational risk factors and systemic contributors.

## Case Description

A 59-year-old male professional Rakugo performer was referred to our hospital with a 2-day history of cold sensation and pain in the right middle, ring, and little fingers. On presentation, he showed poor coloration at the distal aspects of these fingers, raising concern for peripheral circulatory insufficiency.

Contrast-enhanced computed tomography performed the same day revealed a pseudoaneurysm of the right ulnar artery at the hypothenar region. Emergency surgical intervention was performed, consisting of pseudoaneurysm excision and arterial reconstruction using an autologous vein graft.

**Figure 1:**
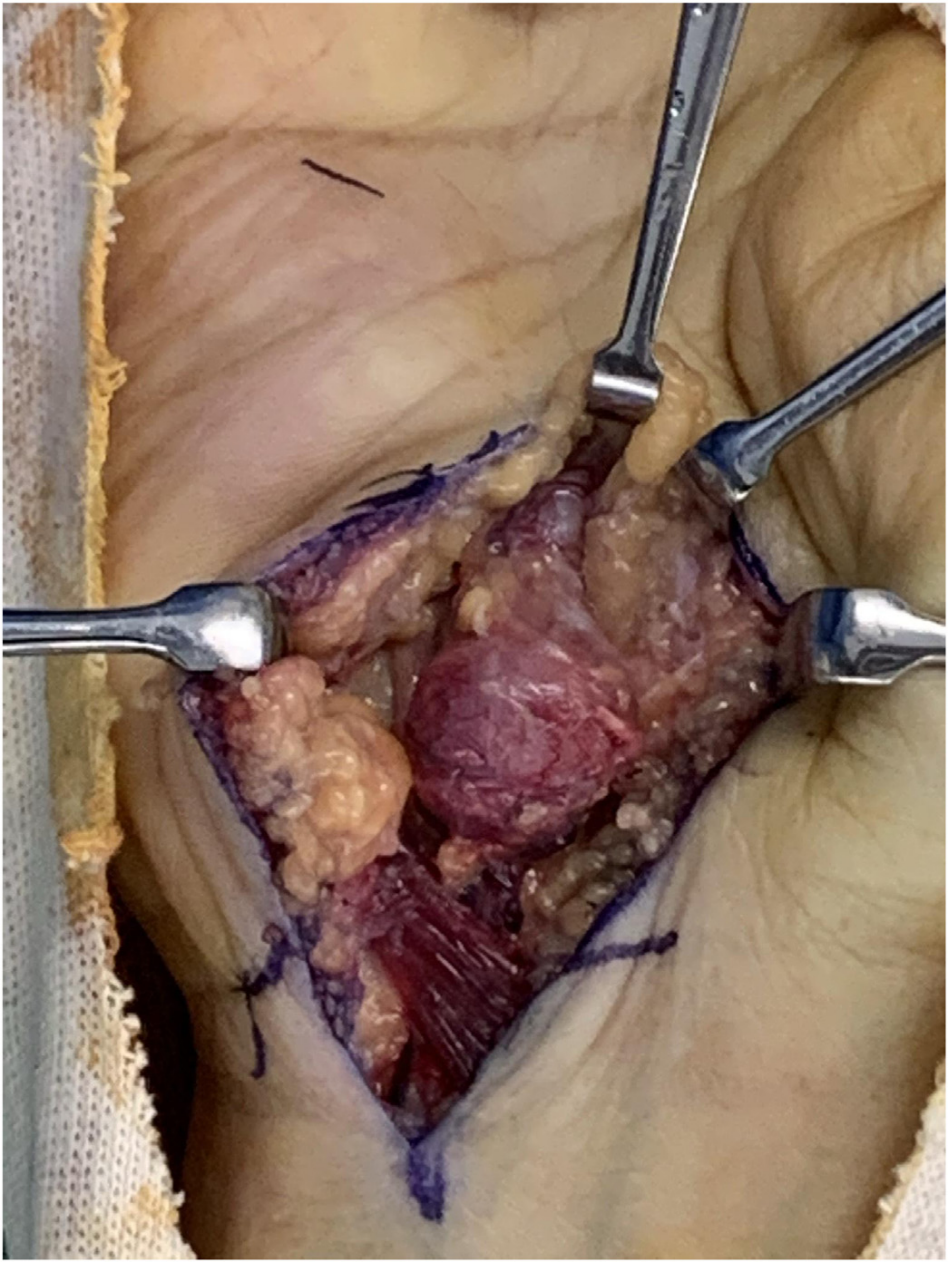
Intraoperative photograph showing a pseudoaneurysm arising from the ulnar artery at the level of the hypothenar eminence. The pseudoaneurysm appears as a well-defined, reddish mass compressing adjacent structures, including the ulnar nerve. The lesion was excised, and arterial reconstruction was performed using a reversed vein graft.

Intraoperatively, the pseudoaneurysm was found to be compressing the adjacent ulnar nerve. The aneurysmal segment was excised, and revascularization was achieved using a reversed superficial vein graft (Fig. 1).

**Figure 2:**
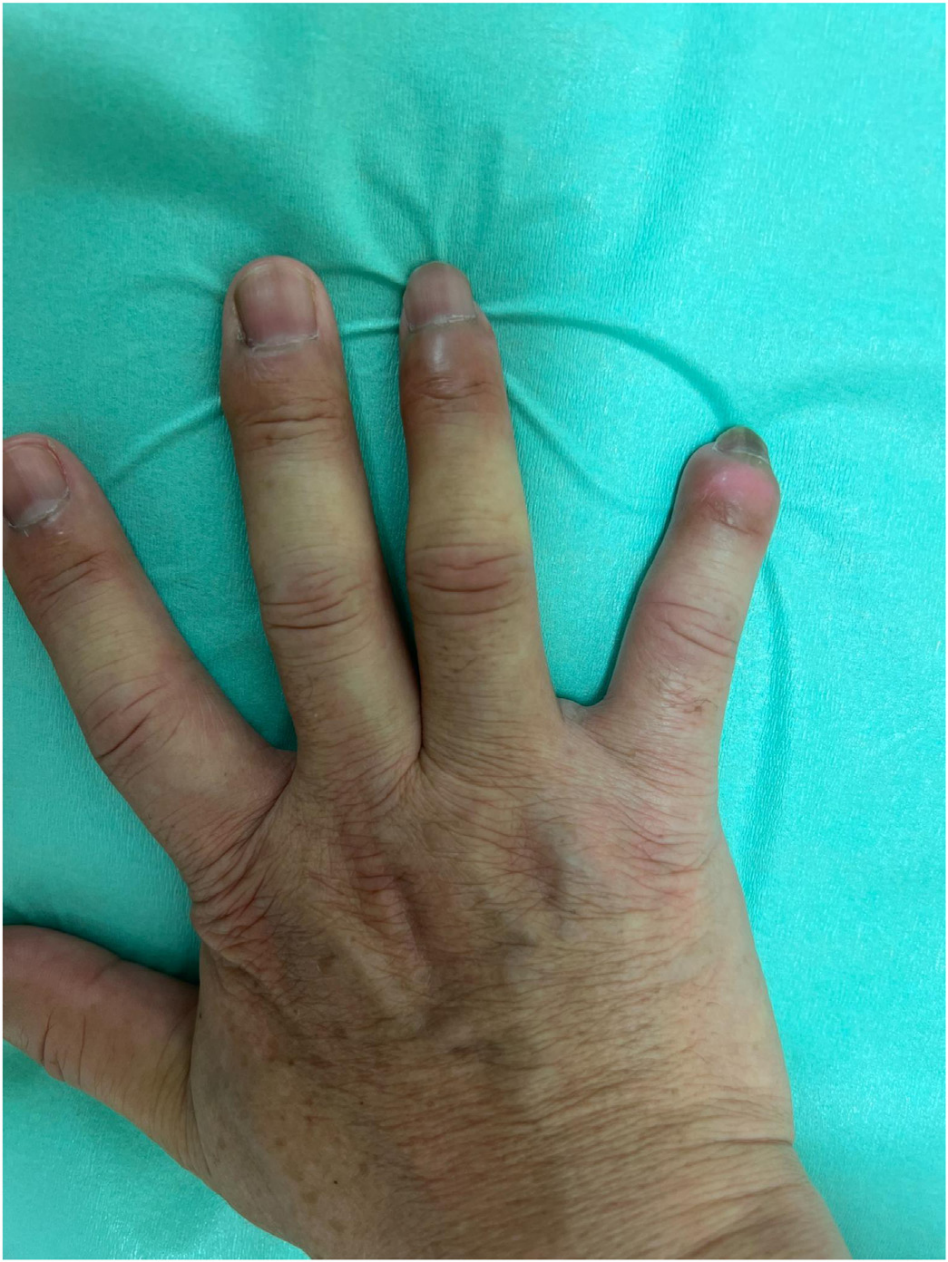
Postoperative photograph taken 6 months after surgery, showing preservation of the distal interphalangeal joints of the right ring and little fingers following fingertip amputations. Functional outcomes were favorable, with adequate range of motion and maintenance of digital length.

Postoperatively, the patient underwent repeated debridement and negative pressure wound therapy. At the 6-month follow-up, the range of motion in the distal interphalangeal joint of the right little finger was 0° of extension to 60° of flexion, and in the ring finger, it was 0° of extension to 90° of flexion. The overall function of the hand was preserved, and the patient was able to return to his professional activities (Fig. 2).

## Discussion

Hypothenar Hammer Syndrome (HHS) is a rare vascular condition caused by repetitive trauma to the ulnar artery at the hypothenar eminence, commonly resulting in arterial wall damage, aneurysm formation, and distal embolization. It typically presents with a chronic and gradually progressive course, manifesting as cold intolerance, digital cyanosis, or exertional pain. Most cases are associated with occupational or recreational activities involving repetitive impact or vibration to the hypothenar area. In the present case, the patient developed sudden digital ischemia of the ulnar-sided digits, despite having remained asymptomatic for over a decade following an initial hand trauma. Imaging revealed a thrombosed ulnar artery pseudoaneurysm at the level of the hypothenar region. The acute onset of ischemic symptoms was likely due to thrombus formation within this long-standing, silent pseudoaneurysm. Although rare, several reports have documented acute presentations of hypothenar hammer syndrome (HHS). Queiroz et al. reported a case of a manual laborer who developed digital ischemia following an acute hyperextension injury, with imaging revealing a thrombosed ulnar artery aneurysm.^1^ Similarly, Jalini et al. described a patient presenting with ulnar artery thrombosis resulting from blunt occupational trauma, leading to acute digital ischemia.^2^ A significant feature of this case is the patient's occupation as a professional Rakugo performer. Rakugo is a traditional Japanese art form of comic storytelling in which performers frequently use a folding fan (sensu) as a prop to mimic various actions. In performance, the fan is often repeatedly struck against the floor (tatami) or other surfaces to accentuate storytelling rhythms or represent physical actions. This repetitive striking motion exerts direct mechanical stress on the hypothenar eminence, particularly when the fan is held firmly and manipulated with force and precision. Such movements are conceptually similar to the repetitive trauma described in HHS patients involved in sports like baseball, tennis, or squash, where the ulnar side of the hand is subject to repeated impact from bats or rackets. Müller et al. reported cases of HHS associated with various sports activities, including baseball, badminton, handball, football, frisbee, softball, karate, weightlifting, and hockey.^3^ Similarly, Zayed et al. described a case of HHS resulting from repetitive trauma during ice hockey stick handling.^4^ However, to our knowledge, there have been no prior reports of HHS in professional Rakugo performers, making this a unique occupational context for the syndrome. Additionally, the ischemic symptoms in our patient developed the day after excessive alcohol consumption, which may have led to dehydration and increased blood viscosity, precipitating acute thrombosis within the aneurysmal segment. Importantly, despite the severity of ischemia, the patient underwent emergency revascularization surgery and required only distal fingertip amputation beyond the DIP joints of the little and ring fingers. This approach preserved overall hand function, and the patient was able to return to his professional activities, representing a favorable functional outcome. This case highlights two clinically important points: first, that HHS, though classically chronic, can present acutely even after a prolonged asymptomatic period; and second, that unique occupational activities such as those in traditional performing arts may pose underrecognized risks for vascular injury. Greater awareness of such possibilities may aid in early recognition and appropriate management of HHS, even in atypical clinical contexts. Written informed consent was obtained from the patient for publication of this case report and accompanying images.

## Conflicts of Interest

No benefits in any form have been received or will be received related directly to this article.
